# Genotyping-by-sequencing based intra-specific genetic map refines a *‘‘QTL*-*hotspot”* region for drought tolerance in chickpea

**DOI:** 10.1007/s00438-014-0932-3

**Published:** 2014-10-25

**Authors:** Deepa Jaganathan, Mahendar Thudi, Sandip Kale, Sarwar Azam, Manish Roorkiwal, Pooran M. Gaur, P B Kavi Kishor, Henry Nguyen, Tim Sutton, Rajeev K. Varshney

**Affiliations:** 1 International Crops Research Institute for the Semi-Arid Tropics (ICRISAT), Hyderabad, India; 2 Osmania University, Hyderabad, India; 3 National Center for Soybean Biotechnology and Division of Plant Sciences, University of Missouri, Columbia, MO USA; 4 Australian Centre for Plant Functional Genomics, School of Agriculture, Food and Wine, University of Adelaide, Waite Campus, Urrbrae, South Australia 5064 Australia; 5 School of Plant Biology and the Institute of Agriculture, The University of Western Australia (UWA), Crawley, WA Australia

**Keywords:** Chickpea, Drought tolerance, Genotyping-by-sequencing, “*QTL*-*hotspot*”, Fine mapping, Candidate genes, CAPS/dCAPS, Marker-assisted breeding

## Abstract

To enhance the marker density in the “*QTL*-*hotspot*” region, harboring several QTLs for drought tolerance-related traits identified on linkage group 04 (CaLG04) in chickpea recombinant inbred line (RIL) mapping population ICC 4958 × ICC 1882, a genotyping-by-sequencing approach was adopted. In total, 6.24 Gb data from ICC 4958, 5.65 Gb data from ICC 1882 and 59.03 Gb data from RILs were generated, which identified 828 novel single-nucleotide polymorphisms (SNPs) for genetic mapping. Together with these new markers, a high-density intra-specific genetic map was developed that comprised 1,007 marker loci spanning a distance of 727.29 cM. QTL analysis using the extended genetic map along with precise phenotyping data for 20 traits collected over one to seven seasons identified 49 SNP markers in the “*QTL*-*hotspot*” region. These efforts have refined the “*QTL*-*hotspot*” region to 14 cM. In total, 164 main-effect QTLs including 24 novel QTLs were identified. In addition, 49 SNPs integrated in the “*QTL*-*hotspot*” region were converted into cleaved amplified polymorphic sequence (CAPS) and derived CAPS (dCAPS) markers which can be used in marker-assisted breeding.

**Electronic supplementary material:**

The online version of this article (doi:10.1007/s00438-014-0932-3) contains supplementary material, which is available to authorized users.

## Introduction

Chickpea (*Cicer arietinum* L.) is the world’s second most important food legume crop, cultivated primarily on marginal lands in the arid and semi-arid regions of South Asia and sub-Saharan Africa. It is a self-pollinated species with basic chromosome number eight and genome size of 738 Mb (Varshney et al. 2013a). Globally, chickpea is cultivated on 13.5 Mha with an annual production of 13.1 Mt (FAO 2013) and is a rich source of protein especially in vegetarian diets. However, chickpea production is affected by various biotic stresses including *Fusarium* wilt, *Ascochyta* blight, *Helicoverpa* and abiotic stresses such as drought, heat and salinity. As chickpea is predominantly cultivated on residual soil moisture, terminal drought is a serious problem and will become more prevalent due to climate change and global warming (Tuberosa 2012; Dodig et al. 2012).

During the past three decades, there has been a shift in cultivation of chickpea from cooler to warmer regions both in Asia and Africa (Kimurto et al. 2014; Krishnamurthy et al. 2013) which has also increased the impact of drought on productivity. Therefore, enhancing the drought tolerance in chickpea would help to stabilize and increase production. Marker-assisted selection (MAS) has already proved its importance in accelerating the process of variety development (Varshney et al. 2005). However, breeding efforts towards developing drought-tolerant chickpea varieties have remained slow, mainly because of precision issues in phenotyping for drought tolerance, narrow genetic base and the limited availability of genomic resources. Nevertheless, in recent years, the availability of large-scale genomic resources (Varshney et al. 2009; Nayak et al. 2010; Gujaria et al. 2011; Thudi et al. 2011; Hiremath et al. 2012) and high throughput phenotyping (Kashiwagi et al. 2013) have facilitated progress towards the genetic analysis of drought tolerance in chickpea. With the increasing efforts, QTLs for drought-related traits have been identified in several studies (Rehman et al. 2011; Hamwieh et al. 2013; Jamalabadi et al. 2013), though their validation has not yet been reported. Recently, Varshney et al. (2014a) reported 45 robust main-effect QTLs (M-QTLs; QTLs which explain >10 % phenotypic variation (PVE) and 973 epistatic QTLs (E-QTLs; explaining 58.2 and 92.19 % PVE), respectively, using two intra-specific RIL mapping populations (ICC 4958 × ICC 1882 and ICC 283 × ICC 8261). In addition, the study also revealed nine QTL clusters including a genomic region on CaLG04 referred to as “*QTL*-*hotspot*”, harboring several QTLs for drought tolerance-related traits. Introgression of this “*QTL*-*hotspot*” in one elite variety, JG 11, has shown improvement of drought tolerance-related traits (Varshney et al. 2013b). However, the “*QTL*-*hotspot*” was genetically large (~29 cM on the genetic map and 7.74 Mb on the physical map; Varshney et al. 2014b) and was associated with relatively few SSR markers making it difficult to identify polymorphism between the recurrent and donor genotypes in a backcrossing strategy and also to identify genes associated with drought tolerance in this region (Thudi et al. 2014). Enriching this region with additional markers will facilitate fine mapping and precision breeding for drought tolerance.

Single-nucleotide polymorphism (SNP) markers have become the markers of choice due to their high abundance and cost efficiency, primarily due to advances in sequencing technologies and their application to genotyping crop species (Silvar et al. 2011). For instance, the genotyping-by-sequencing (GBS) approach proposed by Elshire et al. (2011) has increased the efficiency of SNP discovery and genotyping by enabling high multiplexing of samples and simple library preparation procedures. This approach is now being used in several crops for diversity assessment, trait mapping, genome-wide association studies and genomic selection (Deschamps et al. 2012; Poland and Rife 2012).

In this study, the GBS approach was used to identify and genotype SNPs in an intra-specific mapping population ICC 4958 × ICC 1882 in which the “*QTL*-*hotspot*” region was identified. As a result, several novel SNPs were integrated into “*QTL*-*hotspot*” region and converted to cleaved amplified polymorphic sequences (CAPS) and derived CAPS (dCAPS) markers that can be used cost effectively for molecular breeding to improve drought tolerance in chickpea.

## Materials and methods

### Plant material

One intra-specific recombinant inbred line (RIL) mapping population derived from ICC 4958 × ICC 1882 (ICCRIL03) comprising of 264 individuals was used in the present study. Detailed account on parental lines and mapping population are provided in Varshney et al. (2014a). DNA was isolated from 232 RILs and parental genotypes using high throughput mini-DNA extraction method as described by Cuc et al. (2008). The quality and quantity of DNA were checked using spectrophotometer (Shimadzu UV160A, Japan). Two hundred and eight RILs with high-quality DNA were selected for sequencing.

### Genotyping-by-sequencing (GBS)

A GBS approach was used for SNP calling between the parents and genotyping the RILs as described by Elshire et al. (2011). In brief, the GBS libraries from the parental lines and RILs were prepared using *Ape*KI endonuclease (recognition site: G/CWCG) and sequenced using the Illumina HiSeq 2000 platform (Illumina Inc, San Diego, CA, USA). Genomic DNA of selected mapping population and parental lines were subjected for restriction digestion using endonuclease *ApeK*I for 2 h at 75 °C. Adapters with unique multiplex sequence index (barcodes) were ligated to the sticky ends using ligase buffer with ATP and T4 ligase. Samples were incubated at 22 °C for 1 h and heated to 65 °C for 30 min to inactivate the T4 ligase. Aliquot of each sample (5 µl) was pooled (multiplexed) and purified to remove the excess adapters. DNA samples were eluted in a final volume of 50 µl. PCR was performed to increase the restriction fragments from each library using primers complementary to the corresponding adapters. The amplified pools constituting the “sequencing library,” were cleaned up and evaluated for fragment sizes using a DNA analyzer. Libraries without adapter dimers were subjected to sequencing.

### SNP calling

The reads obtained were first de-multiplexed according to the sample barcodes and adapter sequences were removed using custom perl script. The reads having more than 50 % of low quality base pairs (Phred <5 %) were discarded and filtered data were used for calling SNPs after quality check (Q score >20). The filtered, high-quality data from each sample was aligned to the draft genome sequence (CaGAv1.0) of chickpea (Varshney et al. 2013a) using SOAP (Li et al. 2009). The nucleotide with highest probability at each position under a Bayesian model was identified for individual RILs and the consensus sequences were saved in FASTA format. Consensus sequences from all samples were compared to detect polymorphic loci. Polymorphic loci that were either heterozygous in any of the parents or present in <50 % individuals in the population were discarded and a high-quality SNP dataset was generated.

### Linkage mapping

Genotyping data generated in this and previous studies (See Online Resource 1) were compiled for linkage analysis using JoinMap V4.0 (Van Ooijen and Voorrips 2006). Marker order was assigned using the regression mapping algorithm with maximum recombination frequency of 0.4 at minimum logarithm of odds (LOD) of 3 and jump threshold of 5. Ripple command was used after adding each marker locus to confirm marker order. The Kosambi mapping function was used to calculate the map distance (Kosambi 1943). To detect segregation distortion, Chi-square (*χ* ^2^) values were calculated using Joinmap V4.0. Highly distorted and unlinked markers were excluded from analysis. Mapchart 2.2 (Voorrips 2002) was used to visualize a constructed map for each linkage group. Linkage groups were named according to Varshney et al. (2014a).

### QTL analysis

Genotyping data obtained in the current study and the phenotyping data for 20 drought tolerance-related traits including root traits, morphological, phenological, yield, yield-related traits and drought indices (as mentioned in Varshney et al. 2014a) were used for QTL analysis using QTL Cartographer V.2.5 software (Wang et al. 2012). Composite interval mapping (CIM) was performed by selecting Model 6 with the default window size 10 cM, control marker number 5, and backward regression method. To obtain more precise results the default walk speed was reduced to 1 cM. LOD method (LOD > 3) was used to determine the significance of each QTL interval with the threshold level performed at 1,000 permutations, significance level of *p* ≤ 0.05.

### Conversion of SNPs into CAPS and dCAPS

Single-nucleotide polymorphisms (SNPs) integrated in the “*QTL*-*hotspot*” region were converted to CAPS and dCAPS using dCAPS Finder 2.0 (Neff et al. 2002). The predicted CAPS and dCAPS candidates were amplified in a 20 µl PCR reaction using GeneAmp^®^ PCR System 9700 thermal cycler (Applied Biosystems, Foster City, CA, USA) on 5 parental genotypes of chickpea inter and intra-specific mapping populations (PI 489777, ICC 4958, ICC 1882, ICC 8261 and ICC 283). Amplicons for each CAPS and dCAPS were subjected to digestion using their respective restriction enzymes followed by separation on 2 % agarose gel electrophoresis as described in Gujaria et al. (2011). Details about these primer sequences, PCR conditions and product size are given in Online Resource 2.

### Identification of candidate genes

The amino acid sequences predicted from gene models of genes located in the region delimited by the “*QTL*-*hotspot*” were retrieved from draft genome sequence (CaGAv1.0) of chickpea (Varshney et al. 2013a; http://www.icrisat.org/gt-bt/ICGGC/GenomeManuscript.htm) and searched against NCBI-nr protein database using blast program implemented in Blast2GO software (Conesa et al. 2005) with an E value threshold of ≤e^−20^. Associated gene ontology (GO) terms were exported and searched for plant-related GO terms using the GO slim viewer from the AgBase web server (http://www.agbase.msstate.edu), which also categorize terms into three different classes as biological processes (BP), molecular function (MF) and cellular components (CC).

## Results

### Sequence data and SNP discovery

Parental genotypes of the mapping population (ICC 4958 × ICC 1882) were sequenced at higher depth (5× coverage) than RIL individuals, and a total of 69.39 million reads containing 6.24 Gb for ICC 4958 and 62.79 million reads containing 5.65 Gb for ICC 1882 were generated. In addition, 701.05 million reads containing 59.03 Gb were generated for 208 RILs. The number of reads generated varied from 0.28 million (RIL078) to 19.23 million (RIL204) with an average of 3.37 million per line. The data obtained were filtered and used for SNP identification using SOAP software. The SNPs identified were again parsed to remove heterozygous SNPs in parents and a set of 828 SNPs were identified across 208 RILs. The flanking sequences of all SNPs have been provided in Online Resource 3.

### Construction of genetic map

**Fig. 1:**
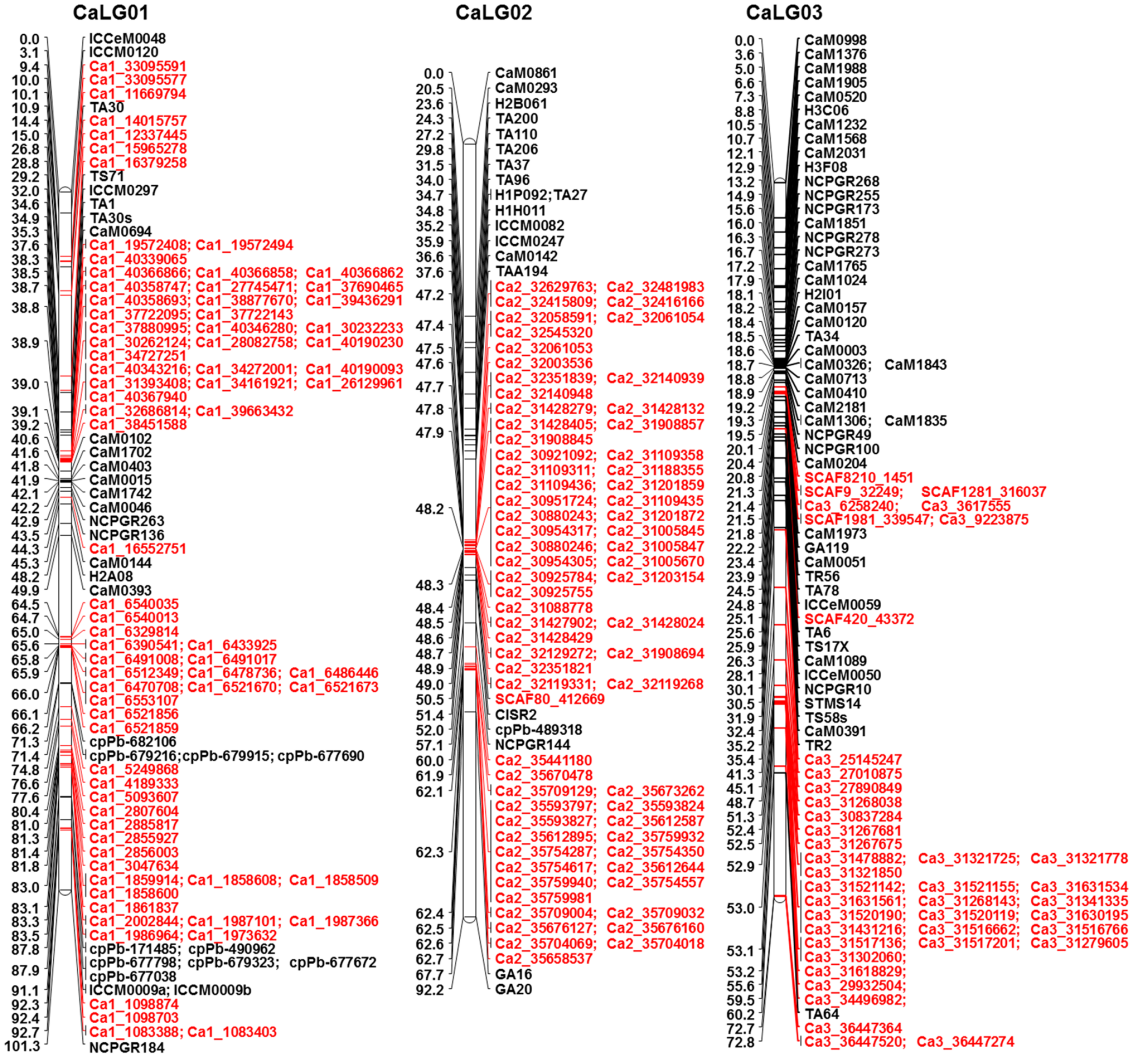

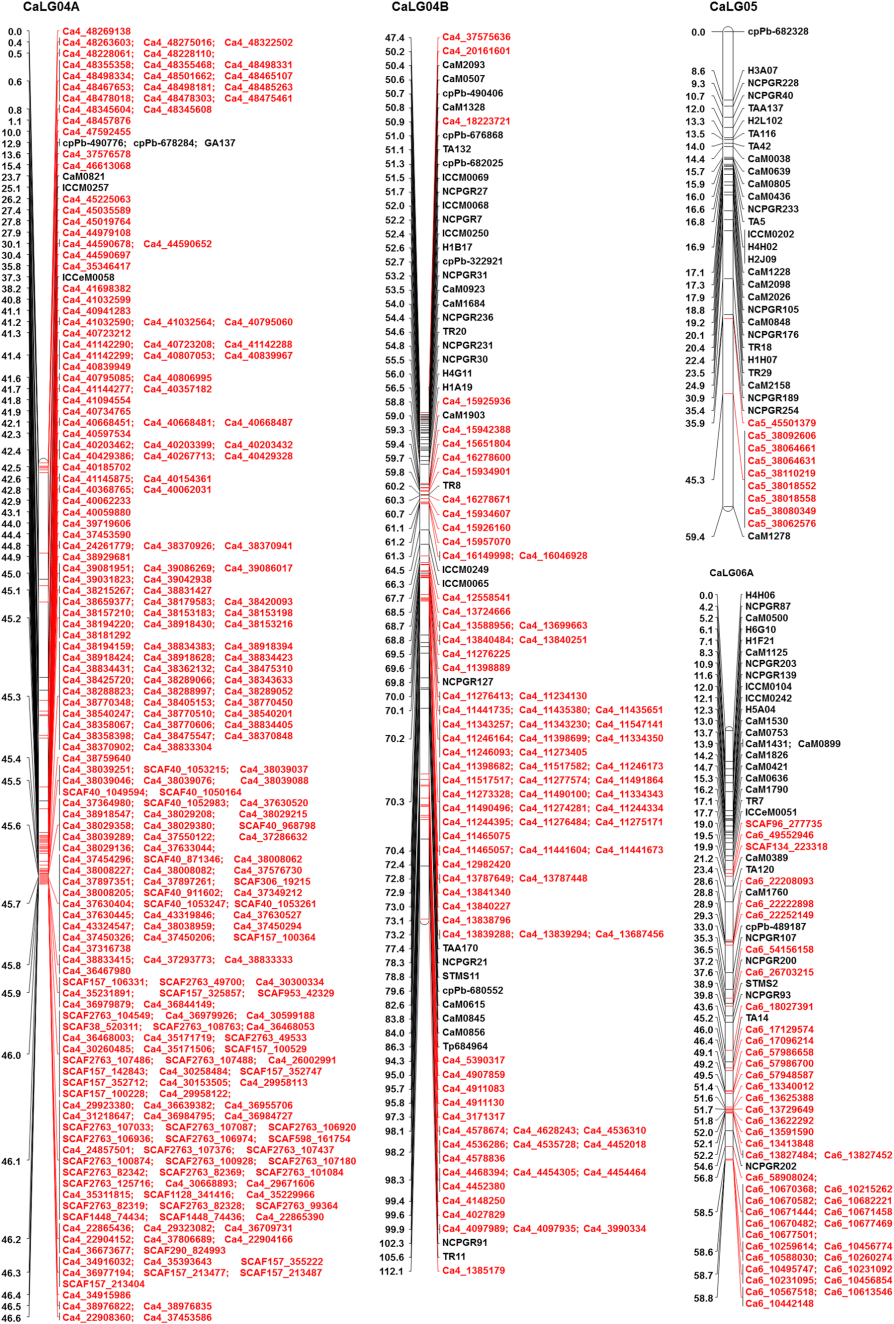

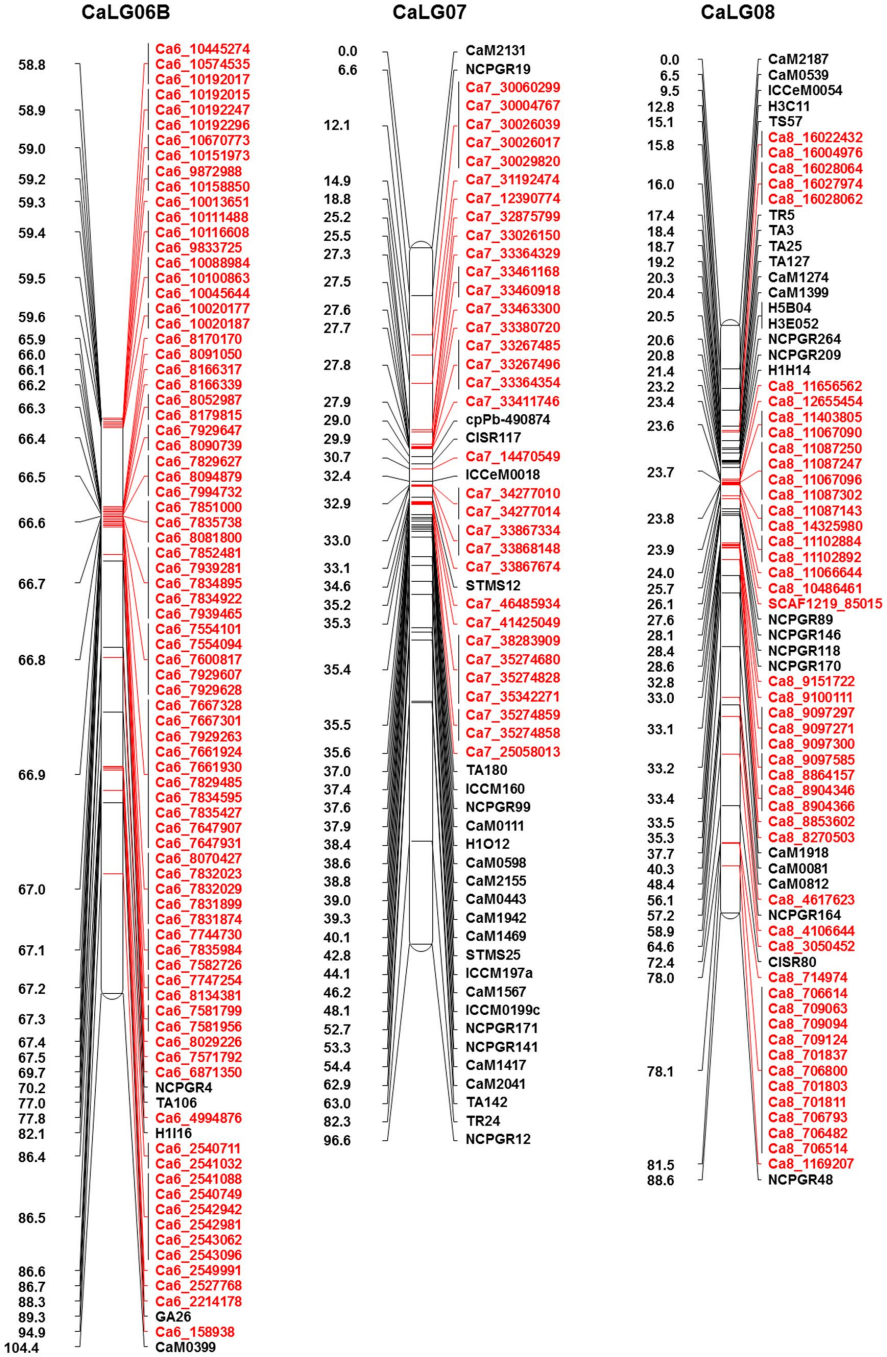
High-density intra-specific genetic map of chickpea (ICC 4958 × ICC 1882). This map is comprised of 1,007 markers including 743 novel SNPs from GBS approach and spans 727.29 cM. Genetic distances (cM) were shown on the *left side* and the markers were shown on the *right side* of the *bars*. Map was constructed using JoinMap 4.0 and Kosambi function. *Markers in black color* font are from the framework map and *markers in red color* font are newly generated SNP markers. For clear visualization, the CaLG04 and CaLG06 were split into two parts and named as A, B

Genotypic data for 828 polymorphic SNPs generated in this study along with 318 markers (including 241 markers from Varshney et al. 2014a) obtained from the earlier studies (Online Resource 1) were used for genetic map construction. In total, 1,146 markers were used for genetic map construction, of which 1,007 (87.87 %) could be mapped on eight linkage groups (CaLG01–CaLG08) covering 727.29 cM (Fig. 1; http://cmap.icrisat.ac.in/cmap/sm/cp/jaganathan/). These included 743 SNPs, 232 simple sequence repeats (SSRs), 21 diversity arrays technology (DArT), 7 expressed sequence tag-SSR (EST-SSR) and 4 genic molecular markers (GMM) (Table 1). The highest number of markers was mapped on CaLG04 (386), while the lowest number of markers was mapped to CaLG05 (39). The distribution of marker loci on 8 linkage groups has been shown in Fig. 1 and Online Resource 4.

**Table 1:** Distribution of different types of markers on the intra-specific genetic map based on the RIL population ICC 4958 × ICC 1882

Marker series	SNP	SSR	EST-SSR	GMM	DArT	Total markers	Distance (cM)	Density (markers/cM)
Total markers used	828	279	14	4	21	1,146		
Total markers mapped	743	232	7	4	21	1,007		
Percent mapped	89.73	83.15	50	100	100	87.87		
Markers unlinked	85	47	7	0	0	139		
Percent unlinked	10.3	16.85	50	0	0	12.13		
*Markers mapped on different linkage groups*								
CaLG01	77	21	1	–	10	109	101.27	1.08
CaLG02	70	18	–	1	1	90	92.16	0.98
CaLG03	41	47	2	–	–	90	72.78	1.24
CaLG04	342	35	1	1	7	386	112.10	3.44
CaLG05	9	29	–	–	1	39	59.41	0.66
CaLG06	124	34	1	–	1	160	104.36	1.53
CaLG07	33	24	1	1	1	60	96.59	0.62
CaLG08	47	24	1	1	–	73	88.62	0.82
Total	743	232	7	4	21	1,007	727.29	
Average						125.88	90.91	1.30

The length of the linkage groups varied from 59.41 cM (CaLG05) to 112.10 cM (CaLG04). The highest marker density was observed for CaLG04, which had 3.44 markers per cM on average, whereas lowest marker density was observed for CaLG07, which had 0.62 markers per cM on average. Overall, the map had 1.30 markers per cM on average (Table 1; Fig. 1). Of 828 SNPs used for linkage map construction, 743 SNPs (89.73 %) were mapped, among which 342 were mapped on CaLG04. Of 279 SSR markers used, 232 (83.15 %) were mapped. Comparatively, SSR markers were mapped evenly on all the eight linkage groups, the highest number of SSR markers was mapped on CaLG03 (47), and the lowest number of SSR markers was mapped on CaLG02 (18). Out of 14 EST-SSRs, 50 % were mapped, whereas all GMM and DArT markers used in the present study were mapped. However, among 21 DArT markers mapped, 47.6 % (10) were on CaLG01, 33.33 % (7) on CaLG03, one each on CaLG02, CaLG05, CaLG06 and CaLG07. Out of four GMM markers, one each was mapped on CaLG02, CaLG04, CaLG07 and CaLG08.

### Marker enrichment in the “*QTL*-*hotspot*” region

**Fig. 2:**
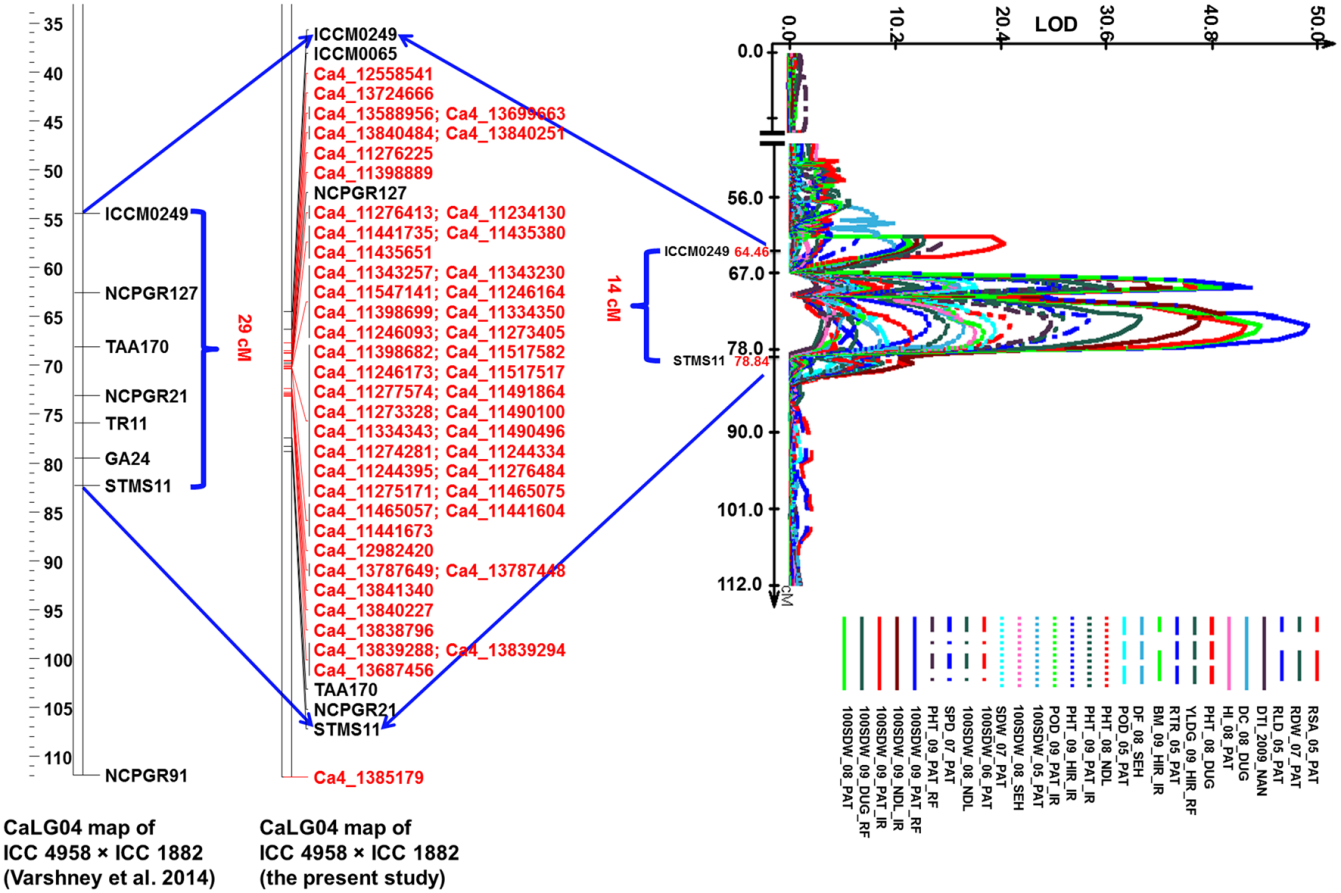
Saturated “*QTL*-*hotspot*” region with additional markers. The figure shows comparison of the “*QTL*-*hotspot*” updated with 49 novel SNP markers in this study and with the one reported by Varshney et al. (2014a)

QTL analysis based on genotypic data for 1,007 markers and phenotypic data for 20 traits (as described in Varshney et al. 2014a), identified a total of 164 robust main-effect QTLs (M-QTLs) by QTL Cartographer 2.5. More than 50 % (91 M-QTLs) of these M-QTLs were located on CaLG04 and significantly, all 91 QTLs were detected in the “*QTL*-*hotspot*” region (Online Resource 5). The earlier reported “*QTL*-*hotspot*” region (Varshney et al. 2014a) had 7 SSR markers (ICCM0249, NCPGR127, TAA170, NCPGR21, TR11, GA24 and STMS11) and spanned 29 cM on linkage group CaLG04. The current study integrates 49 new SNP markers in the “*QTL*-*hotspot*” region spanning 14 cM (Fig. 2).

### QTL analysis

Out of 20 traits analyzed, QTLs were identified for 16, including root length density (RLD, cm cm^−3^), root surface area (RSA, cm^2^), root dry weight/total plant dry weight (RTR, %), shoot dry weight (SDW, g), plant height (PHT, cm), primary branches (PBS), days to 50 % flowering (DF), days to maturity (DM), 100-seed weight (100 SDW, g), biomass (BM, g), harvest index (HI, %), pods/plant (POD), seeds/pod (SPD), yield (YLD, g), drought susceptibility index (DSI) and drought tolerance index (DTI). QTL nomenclature was adopted as per Varshney et al. (2014a). In case a QTL reported for a given trait in (Varshney et al. 2014a) has been further resolved into two or more QTLs, the QTLs are further named using a decimal followed by a roman numeral. For instance *QR3rld01* reported earlier was resolved into three, hence named as *QR3rld01.1*, *QR3rld01.2*, *QR3rld01.3* (Online Resource 5). QTLs were considered as ‘stable’ (if they appeared in more than one location for the specified trait) and ‘consistent’ (if they appear in more than 1 year/season for the specified trait) as described in Varshney et al. (2014a). Identified QTLs are discussed below.

### Root-related traits

Three QTLs were identified, one each for RLD, RSA and RTR with PVE ranging from 10.65 to 13.56 % (Table 2). Among them, RLD and RTR were identified in the “*QTL*-*hotspot*” as reported earlier, whereas a QTL for RSA was identified on CaLG06 (Online Resource 5). The QTL for RLD, ‘*QR3rld01*’ was refined to 3.23–5.37 cM from 10.54 cM, whereas that for RTR, ‘*QR3rtr01*’ was refined to 1.81–5.37 cM from 5 cM (Table 2, Online Resource 5). Both QTLs were consistent across years (2005 and 2007).

**Table 2:** Comparison of robust main-effect QTLs (M-QTLs) identified for various drought tolerance-related traits in the present study with that of Varshney et al. (2014a)

Varshney et al. (2014a) (ICC 4958 × ICC 1882)							Current study (ICC 4958 × ICC 1882)					
Trait	Total QTLs	No. of QTLs in the “*QTL-hotspot*”	Stable QTLs	Consistent QTLs	Position on the genetic map (cM)	PVE^a^ (%)	Total QTLs	No. of QTLs in the “*QTL-hotspot*”	Stable QTLs	Consistent QTLs	Position on the genetic map (cM)	PVE^a^ (%)
**Root**												
RLD	1	1	–	–	10.54	10.90	1	1	–	1	3.23–5.37	10.65–12.09
RSA	1	1	–	–	13.86	10.26	1	–	–	–	7.57	11.04
RTR	1	1	–	–	5.00	16.67	1	1	–	1	1.81–5.37	10.85–13.56
**Morphological**												
SDW	1	1	–	1	5.00	13.89–17.59	3*	3	–	1	0.73–5.91	10.78–26.91
PHT	4	1	1	2	0.96–42.39	10.00–30.20	9*	3	3	5	1.05–17.94	10.05–34.57
PBS	–	–	–	–	–	-	1*	-	-	-	8.81	12.92
**Phenological**												
DF	2	1	1	1	5.53-22.86	10.51-26.87	3*	1	1	1	1.81-15.13	10.86-67.71
DM	3	1	1	1	5.53-31.09	12.13-19.71	2	1	1	1	5.14-15.13	10.11-47.43
**Yield related**												
100SDW	2	1	1	1	10.53-16.65	10.31-58.20	2	1	2	2	1.81-15.41	10.12-60.41
BM	2	1	-	-	10.54-22.86	10.95-21.32	3*	1	-	-	1.81-15.13	10.11-16.63
HI	3	1	-	-	5.00-20.23	10.67-14.36	3	1	-	1	1.81-16.29	10.14-25.94
POD	1	1	-	1	10.54	10.19-23.18	2*	2	-	1	0.86-5.37	10.73-32.34
SPD	1	1	-	-	5.00	42.07	3*	3	-	-	1.81-4.96	11.09-45.40
YLD	2	1	-	-	1.92-5.00	13.98-15.71	3*	1	-	-	2.08-13.44	11.67-18.64
**Drought indices**												
DSI	-	-	-	-	-	-	1*	-	-	-	6.28	13.00
DTI	1	-	-	-	28.90	11.23	3*	1	-	-	1.81-16.29	10.10-10.76
**Total**	25	13	4	7			41	20	7	14		

* Newly identified M-QTLs

^a^ *PVE* phenotypic variation explained

### Morphology-related traits

A total of 3 and 9 QTLs were identified for SDW and PHT, respectively, out of which 2 and 5 were newly identified for the respective traits. Overall, PVE ranged from 10.05 to 34.57 % (Table 2). The QTL size for SDW ‘*QR3sdw01*’ was similar as reported earlier, whereas QTL for PHT, ‘*QR3pht03*’ was refined to 1.81 cM from 5.37 cM (Online Resource 5). Out of 9 QTLs identified for PHT, 3 were stable and 5 were consistent. Interestingly, two QTLs, ‘*QR3pht06*’, and ‘*QR3pht08*’ were found consistent and stable, which were previously reported to be unstable and inconsistent by Varshney et al. (2014a). A QTL for primary branches (PBS) ‘*QR3pbs02*’ was newly identified in the current study which explained the PVE of 12.92 % (Table 2 and Online Resource 5).

### Phenology-related traits

For phenological traits, 3 and 2 QTLs were identified for DF and DM, respectively. The maximum phenotypic variation explained by the QTL, ‘*QR3df04*’ was much higher (67.71 %) as compared to the earlier study (26.87 %) for DF (Online Resource 5). This QTL has been refined to 1.81 cM from 5.14 cM. Similarly, the QTL, ‘*QR3dm01*’ explained 47.43 % PVE for DM which was comparatively higher than that reported earlier (19.71 %) and was refined to 7.33 cM from 15.13 cM (Table 2 and Online Resource 5).

### Yield-related traits and drought indices

A total of 16 QTLs including 5 novel QTLs were identified for yield and yield-related traits, including 2 each for 100 SDW and POD and 3 each for BM, HI, SPD and YLD. Overall, the QTLs spanned the same size as reported previously (Varshney et al. 2014a); however, QTLs for BM and POD have been refined to 15.13 and 5.37 cM, respectively (Table 2). The PVE by each QTL was comparatively high, especially for 100SDW, ‘*QR3100sdw03,*’ which had PVE of 60.41 % (Online Resource 5).

In the case of drought indices, a novel QTL, ‘*QR3dsi02’*, was identified explaining 13.00 % phenotypic variation for DSI, whereas no QTL for DSI was reported in the earlier study (Varshney et al. 2014a) (Table 2). Three QTLs were identified for DTI, of which 2 were novel. Interestingly, a QTL from the “*QTL*-*hotspot*”, ‘*QR3dti02*’, which was earlier reported to be a minor QTL, was ranked as robust in this study (Table 2 and Online Resource 5).

### CAPS and dCAPS marker assays

As breeders are interested in an inexpensive and technically less demanding genotyping platform for marker-assisted breeding, the SNPs integrated into the “*QTL*-*hotspot*” were converted to CAPS/dCAPS markers. A total of 16 CAPS and 33 dCAPS primer pairs were designed and verified for amplification (Online Resource 2). However, only 20 out of 49 primer pairs showed single prominent amplicon and subsequently used for restriction digestion on a panel of 5 parental genotypes. As a result, 14 CAPS and 1 dCAPS were developed (Online Resource 6). In total, 8 CAPS markers were polymorphic in inter-specific mapping population PI 489777 × ICC 4958, while 14 (13 CAPS and 1 dCAPS) each in two intra-specific mapping populations ICC 4958 × ICC 1882 and ICC 283 × ICC 8261 were polymorphic (Online Resource 2).

### Selection of candidate genes

A detailed analysis of QTLs from the “*QTL*-*hotspot*” region showed that, QTLs for 9 traits (RTR, SDW, PHT, DF, 100SDW, DM, HI, SPD and DTI) were flanked by Ca4_11276225 and Ca4_12558541 markers. Further, the traits RTR, RLD, PHT, DF, DM, 100 SDW, BM, POD and YLD were flanked by Ca4_13687456 and NCPGR21 markers. As the QTLs for 13 out of 16 traits fall between markers Ca4_11276225 and NCPGR 21 (whose physical position on genome is 14,146,315 bp), the ~3 Mb region between these markers was selected for candidate gene identification (Online Resource 5). The 3 Mb region contained 286 genes. The amino acid sequences for these 286 genes were searched against the NCBI-nr protein database. Of these, 211 sequences were annotated and 1,050 GO terms were obtained (Online Resource 7 and 8). Categorization of these terms into BP, MF and CC showed predominance of stress-related GO terms in BP class, while in MF class GO terms for binding, catalytic, transferase, hydrolyses and kinase activity were predominantly present. Genes having a direct role in stress such as dehydration-responsive element-binding protein (DREB), heat stress transcription protein, thiamine thiazole synthase and few uncharacterized proteins were also identified in the region (Online Resource 7 and 8).

## Discussion

Drought seems to continue to be a serious constraint to chickpea production. Owing to its complex nature, the genetic dissection of drought tolerance into component traits has been challenging. However, comprehensive insights have been provided into component traits by Varshney et al. (2014a). The reported “*QTL*-*hotspot*” required mapping refinement to allow QTL cloning for component trait improvement through molecular breeding. In the present study, efforts were made to saturate this region to facilitate fine mapping.

### SNP markers and linkage mapping

To date, primarily SSR markers have been used for linkage mapping in chickpea intra-specific populations. Although availability of genomic resources has reduced the SSR marker identification span, polymorphism study and further screening is still a time-consuming and labor-intensive process. As a result, most genetic maps remain limited to only a few hundred markers (Radhika et al. 2007; Jamalabadi et al. 2013; Varshney et al. 2014a). We used a GBS approach which has the advantage of simultaneous SNP identification and genotyping. As a result, we identified 828 novel SNPs. Thus, a greater number of markers are now available for this intra-specific population. As compared to GBS studies in other plant species, SNP markers identified in the present study were less (Poland et al. 2012; Sonah et al. 2013). This might be because of variable number of reads generated per RIL (0.28–19.23 million reads) resulting in more missing data points or very stringent SNP calling criterion adopted, for instance SNPs present in <50 % RILs were excluded.

A total of 1,146 markers were used for linkage map construction, out of which 1,007 (87.87 %) markers were mapped which spanned 727.29 cM. This saturated map has approximatively fourfold more markers and increases the marker density from 0.50 to 1.30 per cM as compared to the previous 241 loci map (Varshney et al. 2014a). Nearly, 94.60 % (228) markers from the earlier study (Varshney et al. 2014a), were mapped on the respective linkage groups in the new map, reflecting the higher level of conservation in marker order between the maps. Interestingly, 46 % of the SNP markers were mapped on CaLG04. This may be due to high repeat-rich regions in the case of Ca4 pseudomolecule and it was evident from our earlier studies (Varshney et al. 2013a), that the average SNP density per Kb (7.6) is higher in the case of chickpea “Ca4” psuedomolecule, i.e., the CaLG04. Further, the study also indicated higher diversity level in elite cultivars of chickpea in the case of “Ca4” pseudomolecule (Theta Pi = 2.8180; Theta *w* = 2.2377). High Theta π and Theta *w* are usually associated with repeat-rich regions in genome.

### Refining the “*QTL*-*hotspot*” and developing breeder-friendly markers

The current analysis integrated 49 new SNP markers in the “*QTL*-*hotspot*” region thereby enriching the same from 7 markers to 55 markers (among 7 previously mapped SSRs, two SSR markers GA24 and TR11 could not be mapped; however, ICCM0065 was newly mapped in this region). Integration of these 49 markers has refined the “*QTL*-*hotspot*” region from 29 to 14 cM. Several fine mapping studies earlier have shown that the integration of additional markers has narrowed down the QTL interval. For instance, in the case of rice, Yu et al. (2011) demonstrated that mapping of additional SNP markers not only detected new QTLs but also increased the resolution of the QTLs. Similarly, Silvar et al. (2012) fine mapped the QTLs for powdery mildew resistance by integrating 32 markers in the QTL region in Spanish barley. Likewise, in case of basmati rice, the “aro3-1” QTL was narrowed down to an interval of 390 kb from the earlier reported interval of 8.6 Mb and “aro8-1” QTL was narrowed down to a physical interval of 430 kb (Singh et al. 2007).

The QTL analysis was performed for 20 different traits and 164 robust M-QTLs were detected for 16 traits which included all 14 reported traits from Varshney et al. (2014a). More than 50 % (91) of QTLs were located on CaLG04 and all were detected in the “*QTL*-*hotspot*” region which highlights the importance of this region in drought tolerance mechanism in chickpea. In addition, the current study also identified new QTLs for PBS and DSI which were not detected/reported earlier. Furthermore, some QTLs which were unstable, inconsistent in the earlier study (Varshney et al. 2014a) were identified to be stable and consistent. For instance, five additional QTLs were identified in the case of PHT and one additional QTL each for SDW, DF, BM, POD, SPD and yield (Online Resource 5). Comparatively, the PVE observed for most of the traits was significantly high, indicating robustness of the identified QTLs.

To enhance molecular breeding for introgressing the “*QTL*-*hotspot*”, SNP markers were converted into CAPS/dCAPS. As the SSR markers from the “*QTL*-*hotspot*” showed less/no polymorphism between ICC 4958 and few recurrent chickpea elite cultivars (Thudi et al. 2014), these CAPS and dCAPS markers would be of interest to breeders in marker-assisted breeding programs to introgress the “*QTL*-*hotspot*” region.

### Candidate gene identification

Functional annotation of the candidate genes revealed their role in various abiotic and biotic stress tolerance mechanisms. For instance, dehydration-responsive element-binding protein (DREB) which is a well-known transcription factor involved in abiotic stress including drought tolerance (Liu et al. 1998; Lata and Prasad 2011) was identified in the “*QTL*-*hotspot*” region. Similarly, thiamine thiazole synthase, which was reported to be involved in stress-related mechanisms (Rapala-Kozik et al. 2012) was also identified in the “*QTL*-*hotspot*” region. In addition to these, few trait-specific genes like E3 ubiquitin–protein ligase and TIME FOR COFFE (TIC) were also identified. The E3 ubiquitin–protein ligase activity has been reported to be involved in grain width and weight in rice (Song et al. 2007) while TIC protein has been reported to play role in plant growth, development and circadian clock (Hall et al. 2003; Sanchez et al. 2011; Shin et al. 2013). Shin et al. (2012) has reported a role of TIC in jasmonic acid signaling pathways and in the control of root meristem size in *Arabidopsis*. Loss of this gene was reported to result in reduced root meristem length and cell number (Hong et al. 2014). Therefore, further fine mapping and cloning of genes underlying QTL would unravel the genetics behind drought tolerance in chickpea.

In summary, we implemented GBS approach for developing a high-density linkage map from an intra-specific population in chickpea. The map contains 1,007 loci spanning 727.29 cM and enriching the “*QTL*-*hotspot*” region from 7 markers to 55 markers. Also this study has refined the “*QTL*-*hotspot*” region from 29 to 14 cM on a genetic map corresponding to ~4 Mb on the physical map. The current study also identified the presence of several stress-related candidate genes including DREB in the "*QTL*-*hotspot*" region. Further characterization of these genes will help in identifying the mechanisms of drought tolerance in chickpea. In addition, the CAPS/dCAPS markers developed in this study can be used in marker-assisted breeding program for introgressing the “*QTL*-*hotspot*” into elite cultivar. Further study and marker enrichment of this region will facilitate fine mapping, QTL cloning and help in understanding the mechanism of drought tolerance in chickpea.

## Electronic supplementary material

Below is the link to the electronic supplementary material.

Summary of validation of CAPS/dCAPS markers on 5 parental line of RIL (XLS 27 kb)

Flanking sequences of the newly identified SNP markers and their flanking sequences (XLSX 15 kb)

The electrophoretic profile of CAPS/dCAPS candidates after PCR and restriction with their respective enzymes (XLSX 139 kb)

Gene ontology (GO) analysis of genes identified in the refined QTL-hotspot region (XLSX 37 kb)

Marker genotyping data from the previous studies included with GBS-SNP markers generated in the present study (XLSX 25 kb)

Categorization of GO terms into biological processes, molecular function and cellular component (TIFF 5784 kb)

Robust main-effect QTLs (M-QTLs) identified for various drought-related traits on ICC 4958 × ICC 1882 (XLS 178 kb)

Details of markers mapped on each linkage group and their genetic positions (XLSX 10 kb)
