# Supplementary figures and images for: Genotyping-by-sequencing based intra-specific genetic map refines a ‘‘QTL-hotspot” region for drought tolerance in chickpea
Source: Mol Genet Genomics. 2014 Oct 25;290(2):559–71. doi: 10.1007/s00438-014-0932-3 (PMC4361754; doi:10.1007/s00438-014-0932-3)

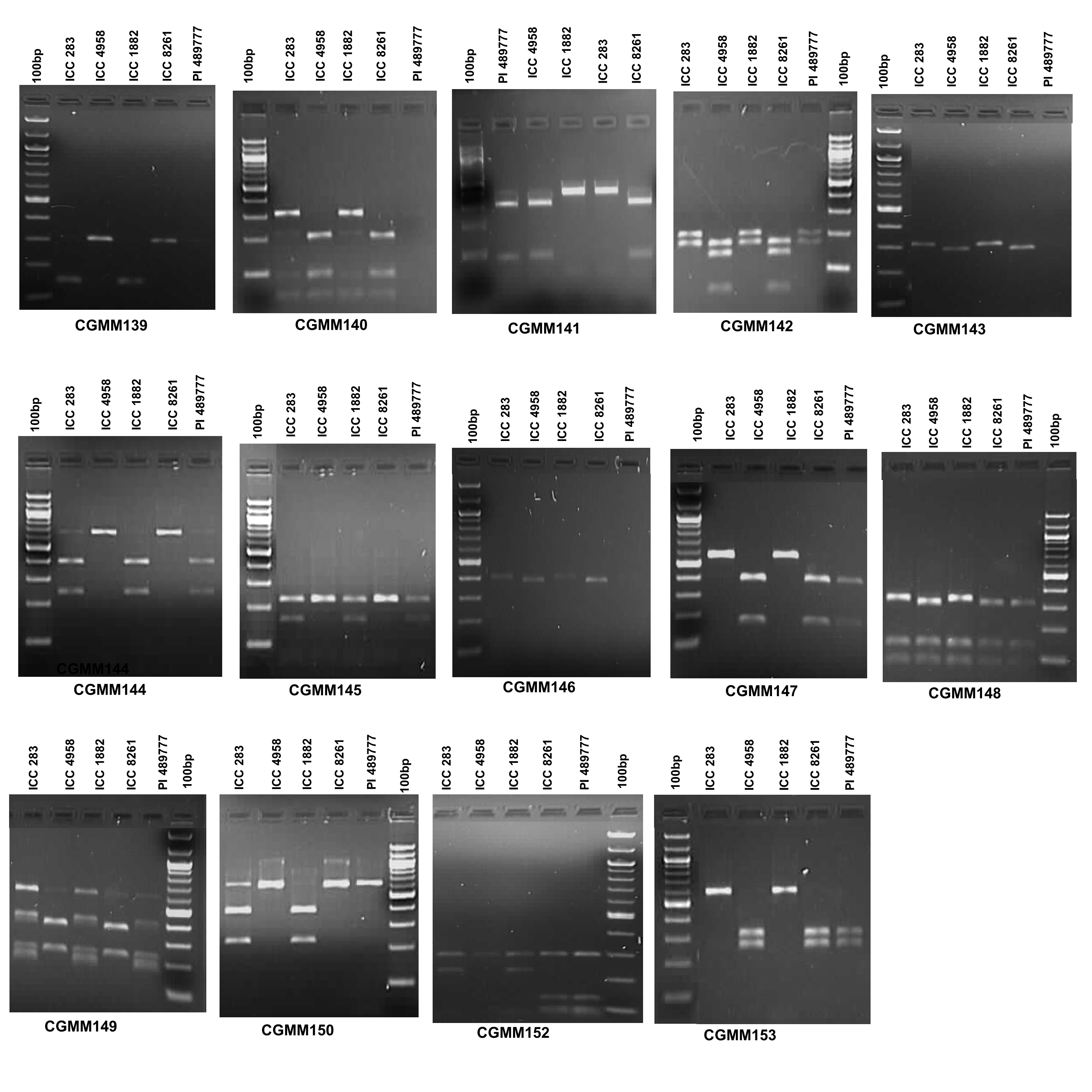

Supplement: Supplementary file 6 — Categorization of GO terms into biological processes, molecular function and cellular component (TIFF 5784 kb) [file 438_2014_932_MOESM6_ESM.tif]
